# Ectopie thyroïdienne: apport du scanner

**DOI:** 10.11604/pamj.2017.26.20.10353

**Published:** 2017-01-17

**Authors:** Traore Ababacar Abdoulaye, Traore Zakaria, Camara Ousmane, Boubbou Meryem, Maaroufi Moustapha, Tizniti Siham, Kamaoui Imane

**Affiliations:** 1 Service de Radiologie du CHU Hassan II Fès, Maroc; 2 Service de Radiologie du CHU Mohammed VI Oujda, Maroc

**Keywords:** Ectopie thyroïdienne, tomodensitométrie, lobe droit, Ectopic thyroidian, tomodensitometric, right lobe

## Abstract

L’ectopie thyroïdienne est une malformation pathologique rare. Nous rapportons un cas supplémentaire d’ectopie du lobe thyroïdien droit, découvert lors du bilan tomodensitométrique d’une masse latéro cervicale gauche.

## Abstract (en)

Ectopic thyroid is a rare malformative disease. We report an additional case of ectopic right thyroid lobe, detected during CT scan evaluation of a left lateral cervical mass.

## Introduction

L’ectopie thyroïdienne est une malformation pathologique rare [1–6]. Elle peut être asymptomatique ou se manifester par une hypothyroïdie clinique ou biologique. L’imagerie est essentielle, car permet de poser le diagnostic [1–6]. Nous rapportons un cas supplémentaire d’une ectopie du lobe thyroïdien droit, découvert lors du bilan tomodensitométrique d’une masse latéro cervicale gauche chez une patiente.

## Patient et observation

**Figure 1:**
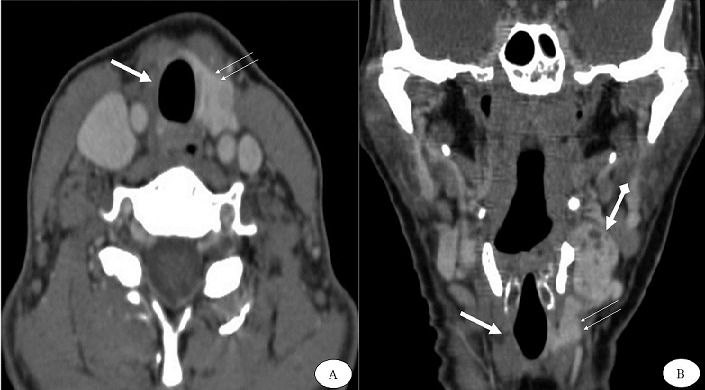
TDM cervicale en coupe axiale (A) et reconstruction coronale (B) après injection de produit de contraste iodé, montrant une loge thyroïdienne droite vide (flèche blanche), un lobe thyroïdien gauche de topographie habituelle normale (double flèche). A noter l’ectopie thyroïdienne gauche (flèche blanche avec tète)

**Figure 2:**
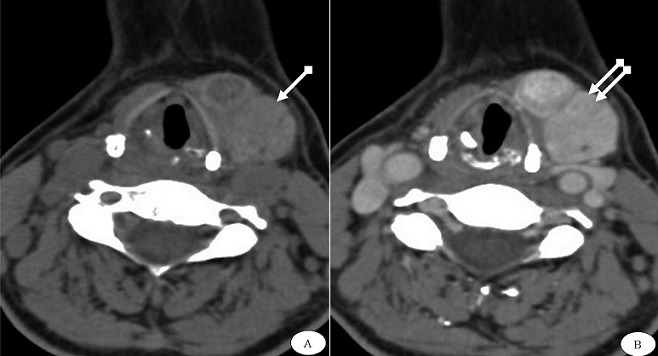
TDM cervicale en coupes axiale en contraste spontané (A) et après injection de produit de contraste iodé (B), montre une masse tissulaire latéro cervicale gauche, de siège para hyoïdien au dessus du lobe thyroïdien gauche, bien limitée, de densité élevée en contraste naturel (flèche blanche) et se rehaussant après injection de produit de contraste iodé (double flèche). Elle observe également des nodules nécrosés en son sein

Patiente de 41 ans, adressée au service pour exploration d’une masse latéro-cervicale gauche. L’histoire de la maladie remonte à une année, par l’apparition d’une tuméfaction latéro cervicale gauche augmentant progressivement de volume, non douloureuse et ne s’accompagnant pas d’autres signes cliniques. L’ensemble de ce tableau évoluait dans un contexte de conservation de l’état général. L’examen physique retrouve une masse latéro cervicale gauche, ferme, indolore, mobile par rapport aux deux plans. La patiente avait un poids à 63 kg pour une taille à 1.59 m, une indice de masse corporelle à 24.9 kg/m^2^, une pression artérielle à 100/80 mm Hg à et un pouls à 58 bats/min. Sur le plan biologique, le bilan hormonal est revenu en faveur d’une hypothyroïdie périphérique avec une TSH à 60µUI/ml et un T4 libre inférieur à 0.4ng/dl. Le reste du bilan est respecté. L’échographie cervicale a montré une masse tissulaire de la région jugulo carotidienne gauche haute, de 30x22 mm de diamètre, faisant évoquée une origine ganglionnaire. Le complément tomodensitométrique cervical objective un lobe thyroïdien gauche de topographie habituelle, mesurant 17x16x30mm (Figure 1). La loge thyroïdienne droite était vide. Le scanner a mis en évidence également une masse tissulaire para hyoïdienne gauche, situé au dessus du lobe thyroïdien gauche, lobulée, bien limitée, de densité spontanément élevée et se rehaussant après injection de produit de contraste iodé. Cette masse mesurait 37x25x41mm de diamètre (Figure 2). Devant cet aspect radiologique qui rappelle un parenchyme thyroïdien, le diagnostic d’une ectopie thyroïdienne du lobe droit est alors retenu. La patiente a bénéficié d’une exérèse chirurgicale, qui a confirmé l’ectopie thyroïdienne gauche en sus lobaire thyroïdienne gauche. L´étude anatomopathologique a confirmé la nature thyroïdienne de la masse tissulaire ectopique gauche, contenant des adénomes trabéculaires, sans signe de malignité tissulaire. Les suites étaient simples avec un recul maintenant de 2 ans.

## Discussion

La thyroïde est une glande cervicale très superficielle accessible à la palpation. Les lobes thyroïdiens droit et gauche sont situés de part et d’autre de la trachée et reliés par l’isthme qui se situe à la jonction tiers moyen-tiers inférieur du lobe [1]. Les anomalies congénitales thyroïdiennes et les variantes de la normale portent sur la taille, la forme, la situation ou la vascularisation [1]. L’ectopie thyroïdienne est une pathologie rare et touche 1/4000-1/8000 des patients atteints d’hypothyroïdie. Elle est deux fois plus fréquente chez les femmes [1–6]. La pathogénie de l’ectopie thyroïdienne reste mal élucider [1–6]. Elle est classiquement sporadique [1]. Cependant, des données récentes suggèrent une composante génétique et familiale d’environ 2% [1]. Dans notre présentation clinique, l’ectopie thyroïdienne serait survenue de manière aléatoire, sans aucune affiliation familiale.

L’étude de l’embryogenèse et du devenir des arcs branchiaux permet d’expliquer à la fois les ectopies du tissu thyroïdien normal et les ectopies intrathyroïdiennes de tissu d’autre origine. Elle se situe qu’en position médiane et paramédiane, et toujours en dedans de l’axe jugulo carotidien [1–7]. Chez l’Homme, la thyroïde dérive d’une ébauche impaire et médiane: l’ébauche thyroïdienne centrale (ETC) et de deux ébauches latérales: les corps ultimobranchiaux. L’ébauche thyroïdienne située au niveau du pharynx primitif forme un bourgeon médian qui migre depuis la base de langue vers le pôle caudal auquel il est relié par le canal thyréoglosse [1–7]. L’ETC aura formé les deux lobes thyroïdiens, l’isthme et éventuellement le lobe pyramidal. À la septième semaine, la thyroïde atteindra sa position définitive [7]. Ainsi du tissu thyroïdien ectopique peut être trouvé le long du tractus thyréoglosse ou au-delà en position médiastinale [1–7]. De ce fait, le tissus thyroïdien ectopique peut être retrouvé en dessous et au dessus de l’os hyoïde, et également au dessus du cartilage thyroïde [8]. Le tissus thyroïdien ectopique peut être sous thyroïdien, réalisant un prolongement d’un pole inferieur ou de l’isthme. Il peut être sans connexion avec la thyroïde et constitue une ectopie vrai [1–7]. Dans les anomalies par insuffisance de migration; la glande est située au dessus de la loge thyroïdienne normale [9]. L’ectopie thyroïdienne peut être asymptomatique ou se manifester par une hypothyroïdie clinique ou biologique [1–6].

L’hypothyroïdie est la principale manifestation clinique de l’ectopie thyroïdienne. Les ectopies de gros volume peuvent échapper au dépistage néonatal, et la sécrétion hormonale résiduelle peut être compatible avec une survie de plusieurs années [10]. Chez notre patiente, le diagnostic d’hypothyroïdie est fait à l’âge adulte. L’orientation du diagnostic positif de l’ectopie thyroïdienne repose tout d’abord sur l’examen clinique, qui vise à palper la loge thyroïdienne et à rechercher une thyroïdienne en situation anormale [5]. Dans notre observation, la patiente présentait une tuméfaction latéro cervicale gauche d’installation progressivement, indolore, sans aucun autre signe clinique associé. A l’âge adulte, le tableau clinique associe souvent un retard statural dysharmonieux, un retard pubertaire, une obésité et un retard psychomoteur d’intensité variable [11]. Les examens d´imagerie sont nécessaires pour la confirmation diagnostic d´ectopie thyroïdienne [1–6]. L’échographie doppler cervicale est l’examen de première intention, elle montre une loge thyroïdienne vide et une masse tissulaire hypo échogène hétérogène en situation anormale [12]. La scintigraphie est une méthode d´exploration fonctionnelle, c’est le gold standard dans l’arbre diagnostique des affections thyroïdiennes notamment malformatives, elle permet de distinguer une athyréose d’une ectopie thyroïdienne et de mettre en évidence un trouble de l’organification [2]. Le scanner permet de poser le diagnostic positif en montrant une masse spontanément dense qui se rehausse après contraste [1–6]. Comme ce fut le cas chez notre patiente. L’IRM analyse mieux les rapports avec les structures adjacentes, et montrent une masse iso ou hyper intense T1, non ou faiblement rehaussé après injection de gadolinium, et hyper intense T2 [7, 8]. Il existe des diagnostics différentiels aux ectopies thyroïdiennes, notamment avec les adénopathies cervicales, les parathyroïdes ectopiques, les pathologies tumorales des glandes salivaires bénignes (adénome, tumeur glomique, schwannome), et malignes (sarcome, carcinome) et les autres anomalies congénitales [12]. Le tableau clinique, le bilan biologique et surtout l’imagerie permettent de guider le diagnostic.

Le traitement médical d’une ectopie thyroïdienne est basé sur l’hormonothérapie substitutive; l’exérèse chirurgicale est indiquée en cas de complication [1]. Chez notre patiente, l’indication opératoire a été posé vu le volume de la masse thyroïdienne. L’évolution a été simple avec une surveillance clinique et échographique périodique. Les complications de l’ectopie thyroïdienne sont le risque d’hémorragie et l’obstruction des voies aériennes supérieures avec dyspnée et ou dysphagie. La dégénérescence maligne reste exceptionnelle (1%), il s’agit essentiellement de carcinome papillaire [13]. En l’absence de traitement, la maladie entraine un déficit intellectuel sévère et une petite taille de la malade [1].

## Conclusion

L’ectopie thyroïdienne par insuffisance de migration est une malformation congénitale rare. Nous rapportons un cas supplémentaire et nous montrons l’intérêt de la tomodensitométrie dans le diagnostic positif de l’ectopie thyroïdienne chez l’adulte.
